# The effect of selenium on the proliferation of bovine endometrial epithelial cells in a lipopolysaccharide-induced damage model

**DOI:** 10.1186/s12917-024-03958-4

**Published:** 2024-03-18

**Authors:** Hanqing Li, Heng Wang, Luying Cui, Kangjun Liu, Long Guo, Jianji Li, Junsheng Dong

**Affiliations:** 1 Department of Clinical Veterinary Medicine, College of Veterinary Medicine , Yangzhou University, Jiangsu Co-innovation Center for Prevention and Control of Important Animal Infectious Diseases and Zoonoses, 12 East Wenhui Rd, Yangzhou, 225009 China; 2 Joint International Research Laboratory of Agriculture and Agri-Product Safety of the Ministry of Education, Yangzhou, 225009 China; 3 International Research Laboratory of Prevention and Control of Important Animal Infectious Diseases and Zoonotic Diseases of Jiangsu Higher Education Institutions, Yangzhou University, Yangzhou, 225009 China

**Keywords:** Selenium, Lipopolysaccharide, Bovine endometrial epithelial cells, Proliferation, PI3K/AKT, Wnt/β-catenin

## Abstract

**Background:**

Endometritis is a common bovine postpartum disease. Rapid endometrial repair is beneficial for forming natural defense barriers and lets cows enter the next breeding cycle as soon as possible. Selenium (Se) is an essential trace element closely related to growth and development in animals. This study aims to observe the effect of Se on the proliferation of bovine endometrial epithelial cells (BEECs) induced by lipopolysaccharide (LPS) and to elucidate the possible underlying mechanism.

**Results:**

In this study, we developed a BEECs damage model using LPS. Flow cytometry, cell scratch test and EdU proliferation assay were used to evaluate the cell cycle, migration and proliferation. The mRNA transcriptions of growth factors were detected by quantitative reverse transcription-polymerase chain reaction. The activation of the phosphatidylinositol 3-kinase (PI3K)/protein kinase B (AKT) and Wnt/β-catenin pathways were detected by Western blotting and immunofluorescence. The results showed that the cell viability and BCL-2/BAX protein ratio were significantly decreased, and the cell apoptosis rate was significantly increased in the LPS group. Compared with the LPS group, Se promoted cell cycle progression, increased cell migration and proliferation, and significantly increased the gene expressions of *TGFB1*, *TGFB3* and *VEGFA*. Se decreased the BCL-2/BAX protein ratio, promoted β-catenin translocation from the cytoplasm to the nucleus and activated the Wnt/β-catenin and PI3K/AKT signaling pathways inhibited by LPS.

**Conclusions:**

In conclusion, Se can attenuate LPS-induced damage to BEECs and promote cell proliferation and migration in vitro by enhancing growth factors gene expression and activating the PI3K/AKT and Wnt/β-catenin signaling pathways.

**Supplementary Information:**

The online version contains supplementary material available at 10.1186/s12917-024-03958-4.

## Background

As an economic animal, the rapid endometrial repair allows dairy cows to enter the next breeding cycle to produce more economic value. After parturition, with damage to the external physical barrier and dilation of the cervix, 80 to 100% of dairy cows are contaminated with bacteria, and up to 40% will develop metritis or endometritis [1–4]. The uterus is a critical reproductive organ in mammals and is mainly composed of epithelial cells and stromal cells of the endometrium and smooth muscle cells of the peripheral muscle layer [5]. Epithelial cells are the first line of defense against microbial pathogens and uniquely positioned to come into constant contact with bacteria and bacterial products [6]. Normally, the uterus in dairy cows will first remodel the caruncles and regenerate the epithelial cells, followed by deeper layer repair [7]. Therefore, the proliferation of bovine endometrial epithelial cells (BEECs) is essential for the repair of the bovine uterus. However, inflammation in the uterus reduces endometrial epithelial cells viability and enhances apoptosis and inflammatory factors secretion, leading to infertility, false pregnancy, stillbirth or abortion in female animals and even causing diseases of the male reproductive system during mating [8–11]. Gram-negative bacteria such as *Escherichia coli* (*E. coli*) have been identified as the main pathogenic microorganism causing bovine endometritis. Lipopolysaccharide (LPS), as its main virulence factor, has been widely used to establish endometritis models in vitro [12, 13].

Selenium (Se) is an essential trace element for maintaining the normal physiological functions of animals. Se levels were found to be associated with the incidence of placental retention, mastitis and endometritis [14–17]. The ultimate purpose of treating endometritis is to reduce inflammation and promote endometrial repair to enter the next reproductive cycle as soon as possible. Several studies found proliferation effects of Se supplementation on some ruminant cells. In sheep, Se can regulate the proliferation and apoptosis of spermatogonial stem cells through the P21-mediated P53 signaling pathway [18]. Se promoted luteinized granulosa cells proliferation and steroidogenesis in goats by activating the PI3K/AKT and AMPK pathways [19]. In cattle, Se can increase the proliferation and cell viability of bovine mammary epithelial cells and reduce cell apoptosis under oxidative stress [20, 21]. However, the proliferation effect of Se on BEECs is far from being understood.

Currently, antibiotics are the most commonly used treatment for endometritis, but antimicrobial resistance, drug residues and food safety have received increasing attention [22, 23]. In this study, a damage model of BEECs was established using LPS to investigate the effect of Se on cell proliferation and elucidate the related mechanism. This study will be beneficial for exploring a new method for preventing and treating bovine endometritis.

## Results

### Establishment of BEECs damage model

**Fig. 1:**
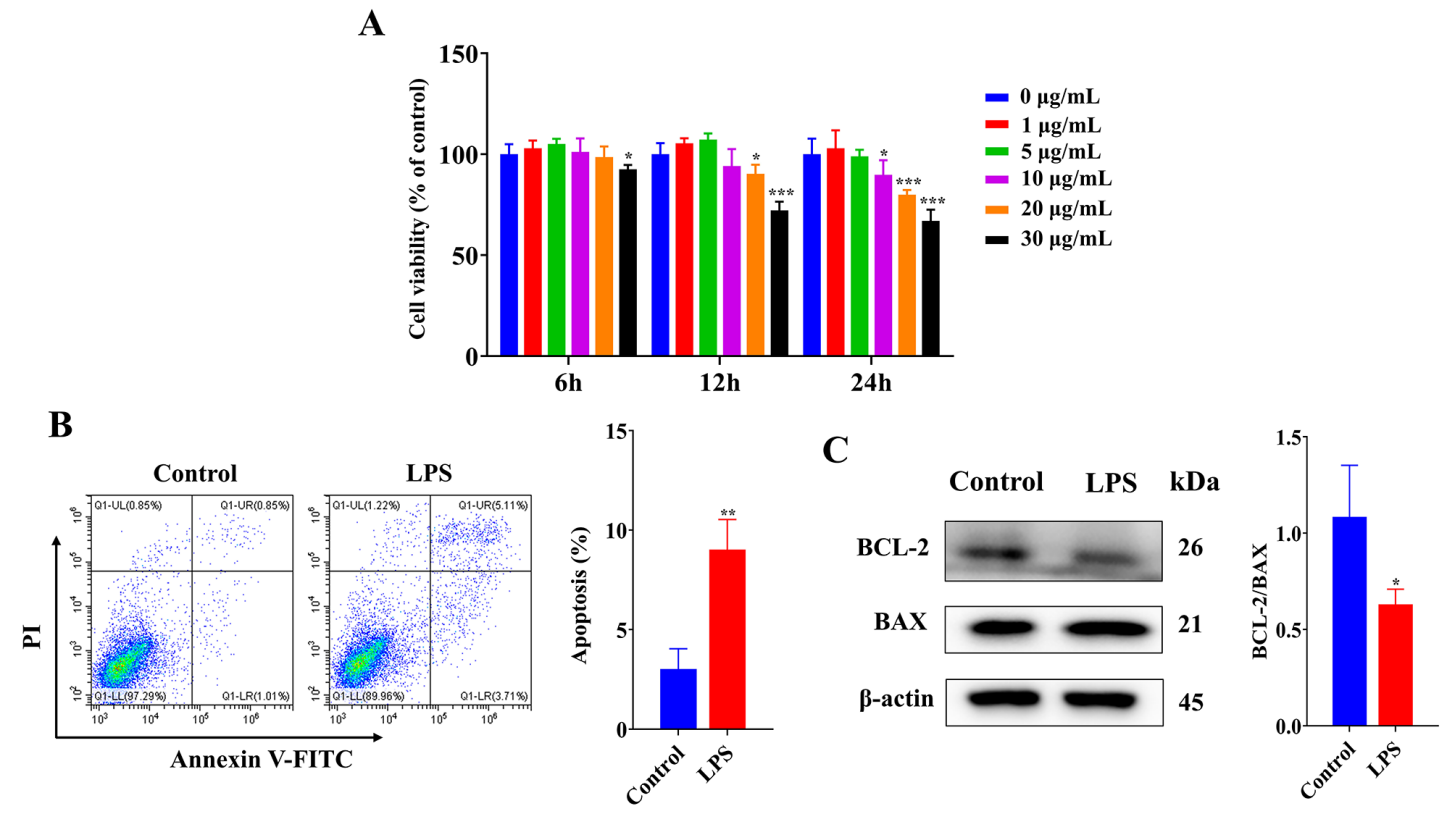
(**A**) The viability of the BEECs following treatment with different concentrations (0, 1, 5, 10, 20 and 30 µg/mL) and times (6, 12 and 24 h) of LPS. The cell viability was measured by CCK-8 method. (**B**) The BEECs were treated with LPS (10 µg/ml) for 24 h and the cell apoptosis was significantly increased. (**C**) The BCL-2/BAX protein ratio was significantly decreased in the LPS group. *, *p* < 0.05, **, *p* < 0.01 and ***, *p* < 0.001 vs. the control group. Data were presented as the mean ± SEM. Original blots were presented in Supplementary Info File 1. The blots were cut prior to hybridization with antibodies. The samples derive from the same experiment and that blots were processed in parallel

The results (Fig. 1A) showed that BEECs viability was reduced (*P* < 0.05 or 0.001) with 30 µg/mL LPS at 6 h, 20 and 30 µg/mL LPS at 12 h and 10, 20 and 30 µg/mL LPS at 24 h. Furthermore, we selected 10 µg/mL LPS treatment for 24 h for follow-up experiments. Compared with the control group, the cell apoptosis rate (Fig. 1B) was increased (*P* < 0.01) and the BCL-2/BAX protein ratio (Fig. 1C) was decreased (*P* < 0.05) in the LPS group. These results indicated that the cell damage model was successfully established.

### Se promotes LPS-inhibited cell migration in BEECs

**Fig. 2:**
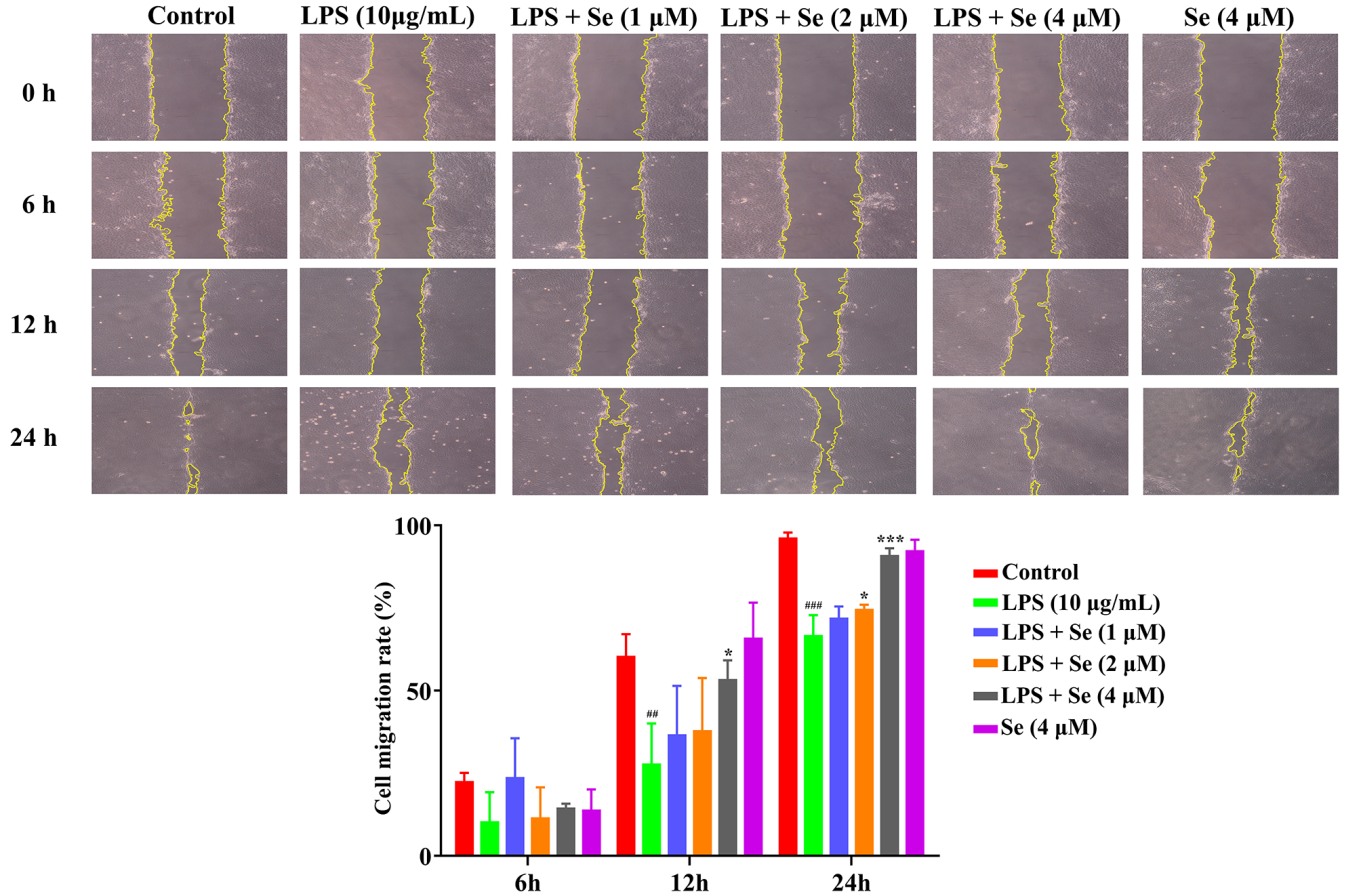
The effect of Se on the cell migration rate of the BEECs by using the scratch wound healing assay. The cells were treated with LPS or Se (4 µM) or co-treated with LPS and Se (1, 2 and 4 µM) and observed under light microscopy at 100× magnification. The yellow lines indicate the cell margins at each time point. The cell migration rate = (scratch width at 0 h – scratch width at 6 or 12 or 24 h)/scratch width at 0 h × 100%. #, *P* < 0.05, ##, *P* < 0.01 and ##, *P* < 0.001 vs. the control group; *, *P* < 0.05, **, *P* < 0.01 and ***, *P* < 0.001 vs. the LPS group. All data were presented as the means ± SEM

As shown in Fig. 2, there was no significant difference in the cell migration rate among the groups at 6 h. Compared with the control group, the cell migration rate was decreased (*P* < 0.01) in the LPS group at 12 and 24 h. Compared with the LPS group, the cell migration rate was positively correlated with the Se concentration and was increased (*P* < 0.01) at 2 and 4 µM.

### Se promoted cell cycle operation and enhanced LPS-inhibited cell proliferation in BEECs

**Fig. 3:**
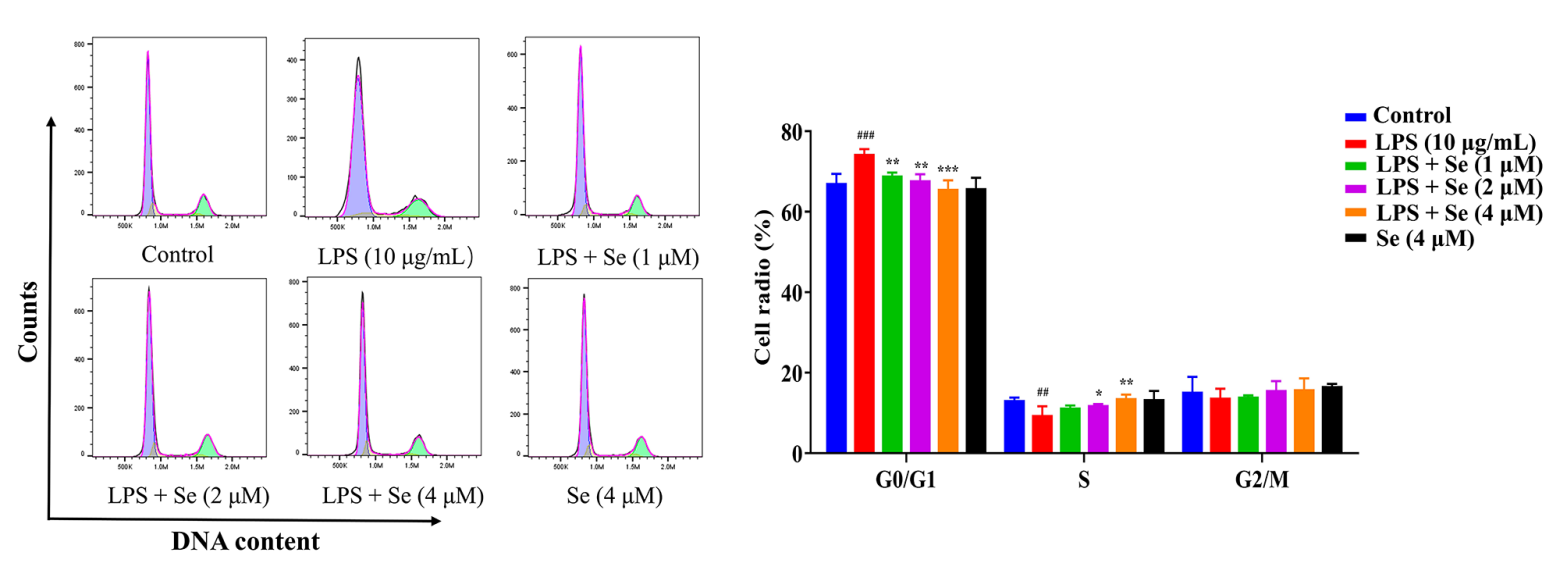
The effect of Se on the cell cycle distribution of the BEECs. The cell cycle distribution was detected by flow cytometry after treated with LPS or Se (4 µM) or co-treated with LPS and Se (1, 2 and 4 µM). #, *P* < 0.05, ##, *P* < 0.01 and ##, *P* < 0.001 vs. the control group; *, *P* < 0.05, **, *P* < 0.01 and ***, *P* < 0.001 vs. the LPS group. All data were presented as the means ± SEM

**Fig. 4:**
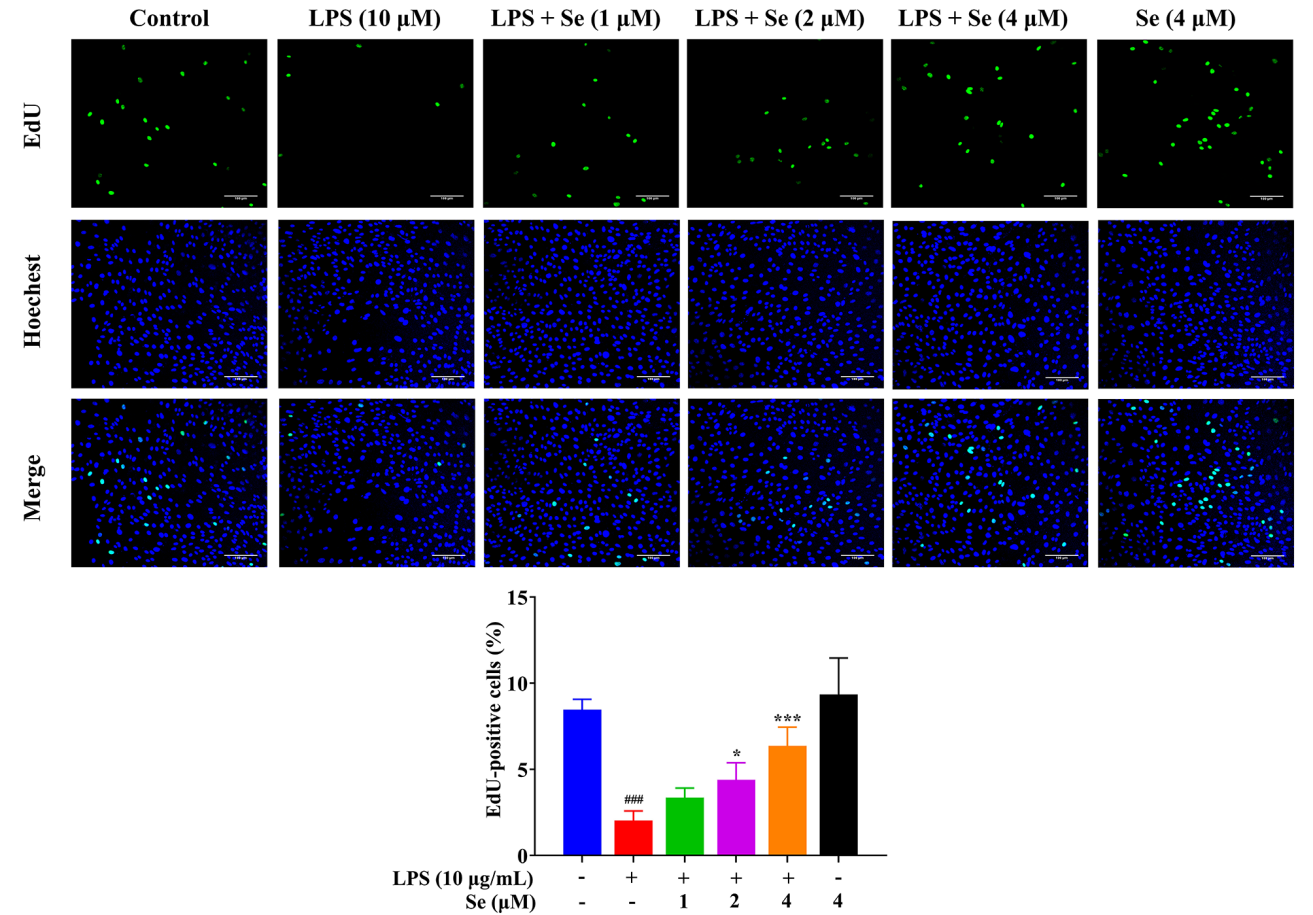
EdU assay of the cell proliferation ability in BEECs. The cells were treated with LPS or Se (4 µM) or co-treated with LPS and Se (1, 2 and 4 µM). #, *P* < 0.05, ##, *P* < 0.01 and ##, *P* < 0.001 vs. the control group; *, *P* < 0.05, **, *P* < 0.01 and ***, *P* < 0.001 vs. the LPS group. The scale bar = 100 μm. All data were presented as the means ± SEM

As shown in Fig. 3, after treatment with 10 µg/mL LPS for 24 h, the number of cells increased (*P* < 0.001) in the G0/G1 phase and decreased (*P* < 0.01) in the G2 phase, indicating that the cell cycle was arrested in the G0/G1 phase. Compared with the LPS group, the number of cells decreased (*P* < 0.01 or 0.001) in the G0/G1 phase and increased (*P* < 0.05 or 0.01) in the G2 phase in the co-treatment groups with LPS and Se, indicating that Se could promote LPS-inhibited cell growth by promoting cell cycle operation. In addition, the results (Fig. 4) showed that the proportion of EdU-positive cells inhibited by LPS showed a dose-dependent relationship with Se concentration. These results imply that Se can enhance the proliferation of BEECs inhibited by LPS.

### Se increased the LPS-inhibited secretion of cell-associated growth factors in BEECs

**Fig. 5:**
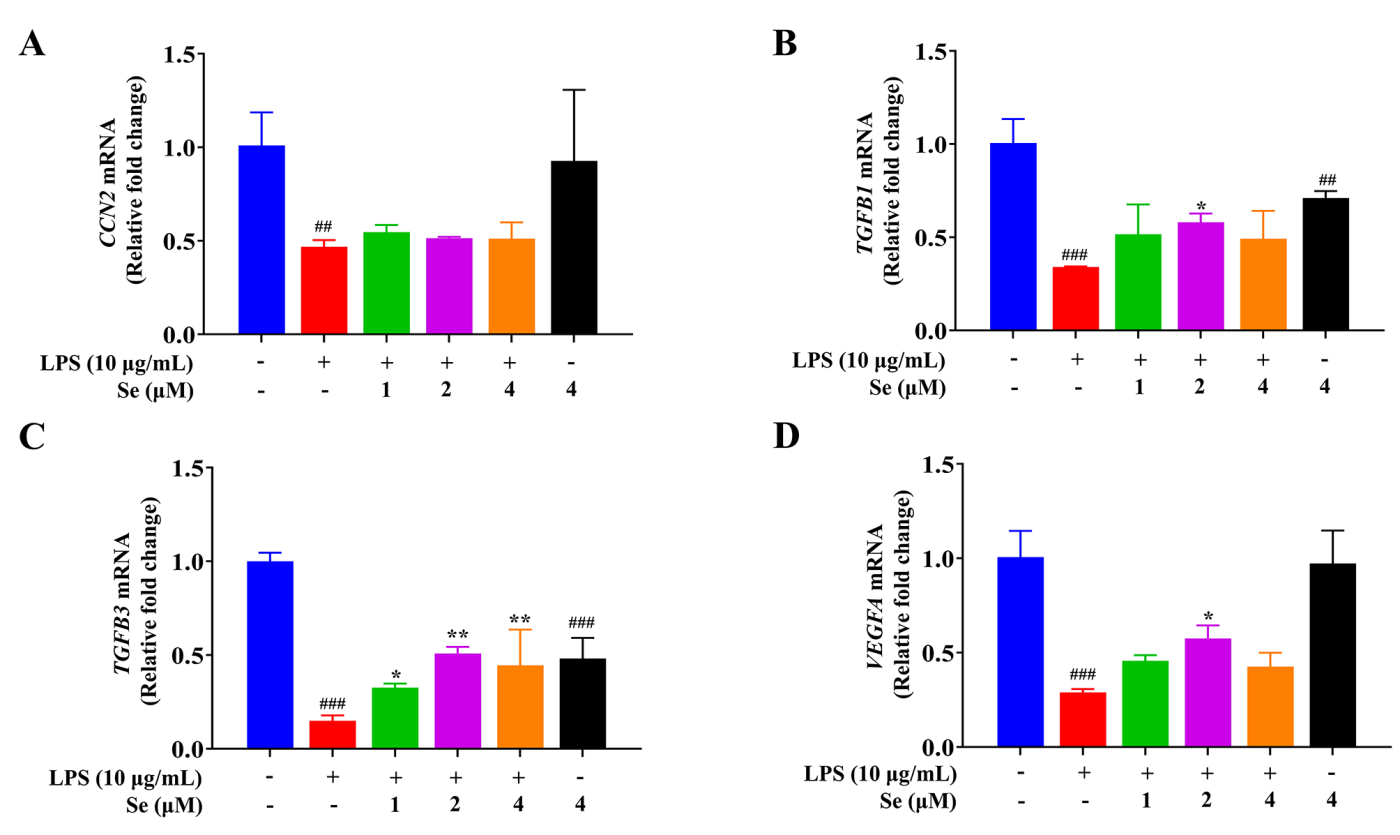
Effects of Se on the gene expressions of *CCN2* (**A**), *TGFB1* (**B**), *TGFB3* (**C**), and *VEGFA* (**D**) in BEECs. The cells were treated with LPS or Se (4 µM) or co-treated with LPS and Se (1, 2 and 4 µM). #, *P* < 0.05, ##, *P* < 0.01 and ##, *P* < 0.001 vs. the control group; *, *P* < 0.05, **, *P* < 0.01 and ***, *P* < 0.001 vs. the LPS group. All data were presented as the means ± SEM

As shown in Fig. 5, LPS treatment decreased (*P* < 0.01 or 0.001) the gene expressions of *CCN2*, *TGFB1*, *TGFB3* and *VEGFA* in BEECs compared with the control group. Compared with the LPS group, the gene expression levels of *TGFB1*, *TGFB3* and *VEGFA* in the co-treatment groups with LPS and Se were increased (*P* < 0.05 or 0.01), but there was no significant difference in the mRNA levels of *CCN2*.

### Se activated the LPS-inhibited PI3K/AKT and Wnt/β-catenin signaling pathways in BEECs

**Fig. 6:**
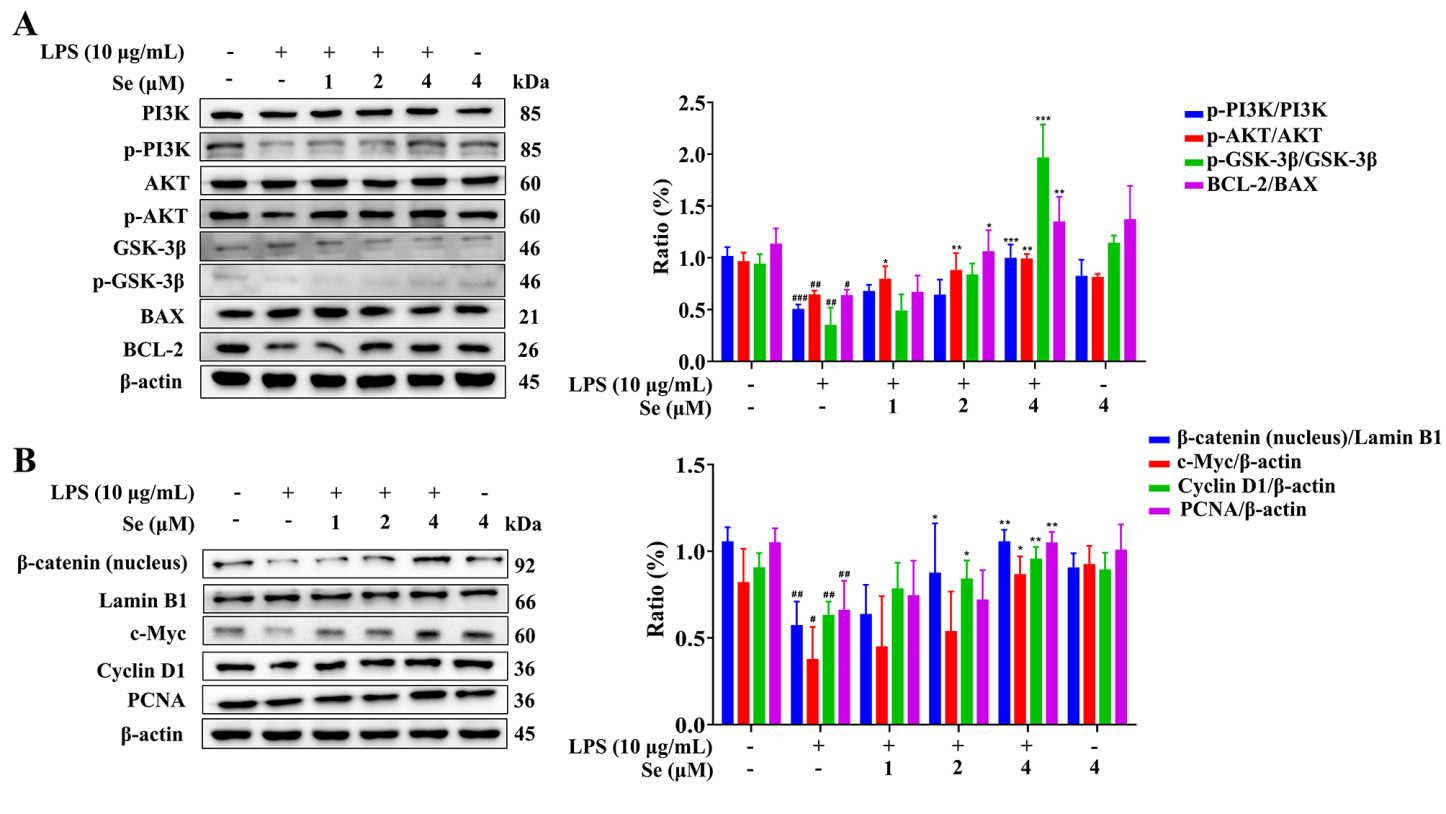
Effects of Se on the PI3K/AKT (A) and Wnt/β-catenin (B) signaling pathways in BEECs. The cells were treated with LPS or Se (4 µM) or co-treated with LPS and Se (1, 2 and 4 µM). #, *P* < 0.05, ##, *P* < 0.01 and ##, *P* < 0.001 vs. the control group; *, *P* < 0.05, **, *P* < 0.01 and ***, *P* < 0.001 vs. the LPS group. All data were presented as the means ± SEM. Original blots were presented in Supplementary Info File 1. The blots were cut prior to hybridization with antibodies. The samples derive from the same experiment and that blots were processed in parallel

The results in Fig. 6 showed that the phosphorylation levels of PI3K, AKT and GSK-3β, the protein levels of β-catenin (nucleus), c-Myc, Cyclin D1 and PCNA, and the BCL-2/BAX protein ratio in the LPS group were decreased (*P* < 0.05, 0.01 or 0.001) compared with those in the control group. These indexes in the co-treatment groups with LPS and Se were dose-dependent with Se concentration and reached the highest value when the Se concentration was 4 µM, which showed an increase (*P* < 0.01 or 0.001) compared with the LPS group.

### Se increased the LPS-inhibited distribution of β-catenin in the nucleus of BEECs

**Fig. 7:**
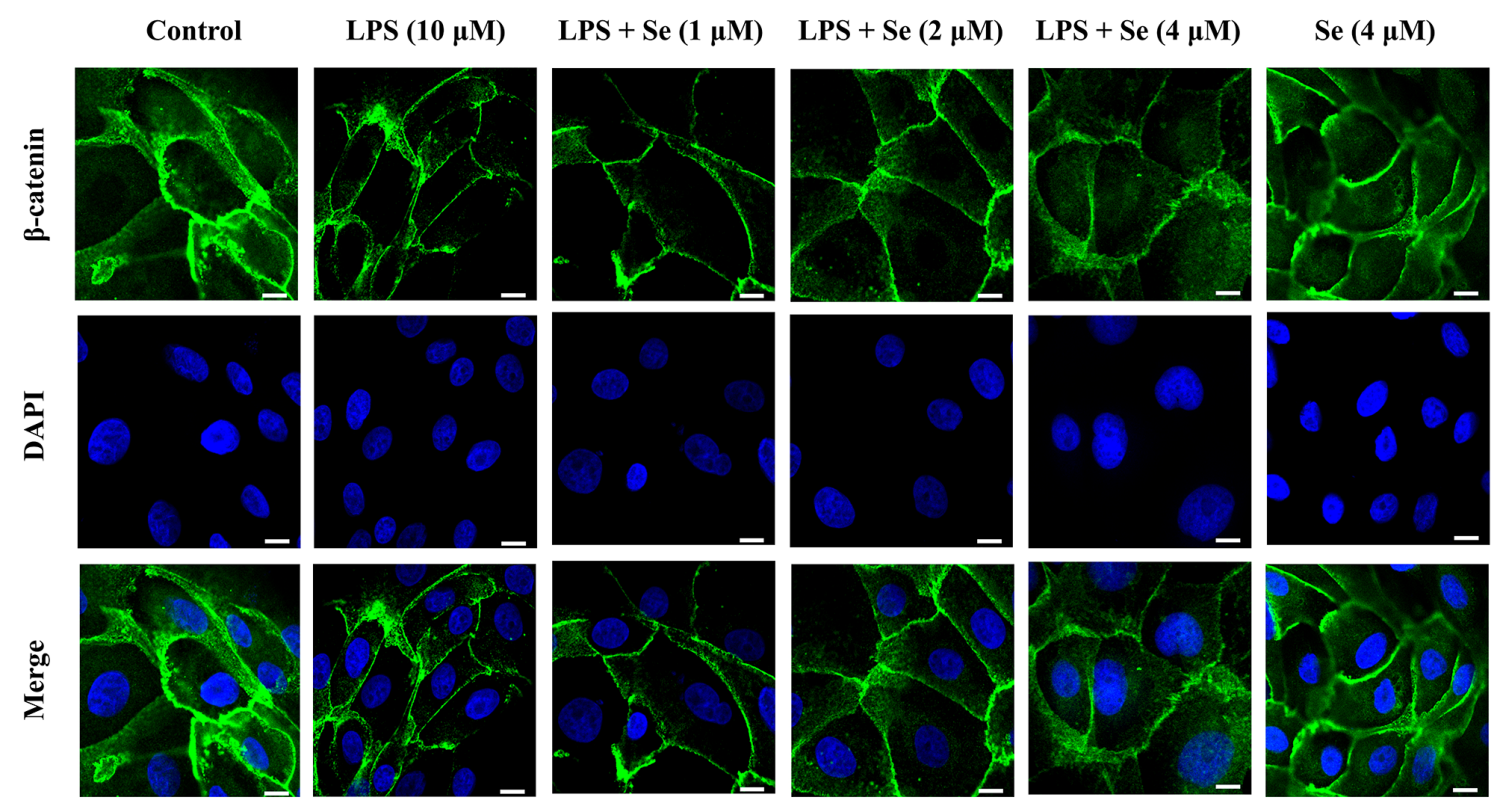
Effects of Se on the nuclear-transport of β-catenin in BEECs. The cells were treated with LPS or Se (4 µM) or co-treated with LPS and Se (1, 2 and 4 µM). The β-catenin levels were evaluated by confocal microscopy. The scale bar = 10 μm. All data were presented as the means ± SEM

As shown in Fig. 7, compared with the control group, the level of β-catenin in the nucleus in LPS group seemed lower. Compared with the LPS group, the β-catenin levels in the nucleus in the LPS and Se co-treatment groups seemed higher and were correlated with the Se concentration.

## Discussion

To our knowledge, this is the first study regarding evidence for the effect of Se on LPS-induced proliferation of BEECs in vitro. Our results suggested that Se protected BEECs from LPS-induced damage and promoted cell proliferation and migration by activating the PI3K/AKT and Wnt/β-catenin signaling pathways and enhancing the expression of some growth factors. Therefore, Se could be a potential therapeutic strategy for female cows with endometritis.

Endometritis is a common disease in dairy cows caused by infection with pathogenic microorganisms, resulting in the decline of fertility and milk production and damaging the normal physiological function of cows [24, 25]. It has been found that there is massive necrosis and apoptosis of BEECs in cows with endometritis [26]. The anti-apoptosis protein BCL-2 and pro-apoptosis protein BAX are closely related to cell apoptosis [27]. The balance between these two protein types controls the mitochondrial apoptotic pathway by regulating mitochondrial permeability to release cytochrome c [28, 29]. Similarly, the cell damage model established in this study also showed that LPS decreased cell viability and the BCL-2/BAX protein ratio and induced apoptosis in BEECs.

Se is an essential trace element for animals and is closely related to normal growth and development in livestock [30]. Dietary Se supplementation could increase the weight of multiple organs (including the uterus) [31, 32]. Our results showed that Se addition to the basal medium promoted the cell cycle, increased the proportion of nuclear proliferation, and accelerated the migration of BEECs under LPS inhibition. Similar to our results, Se could stimulate the granulosa cell and BMSC proliferation in bovine [20, 33]. Se could also promote the migration rate and proliferation of retinal capillary cells and aortal cells [34]. In fact, the effect of Se on cell proliferation in vitro has long been studied. To date, most cell cultures and sera already contain trace amounts (nmol/l) of Se [35].

Tissue repair is a dynamic and sequential process regulated by many factors. Cytokines are extremely potent biomolecules produced by almost all cell types [36]. Vascular endothelial growth factor (VEGF) has mitogenic and anti-apoptotic effects on endothelial cells and is a critical pro-angiogenic factor in the endometrium [37, 38]. Connective tissue growth factor (CTGF), also named CCN2, regulates biological functions such as cell proliferation, migration, adhesion, wound healing, and angiogenesis [39, 40]. Transforming growth factor-beta (TGF-β) controls cellular proliferation, apoptosis, cell migration and adhesion [41]. In this paper, we found that LPS-inhibited mRNA expression levels of these growth factors were increased after Se supplement, although the gene expression level of *CCN2*was not significantly increased. These results suggested that Se can enhance the expression of these growth factors to promote the proliferation of BEECs under LPS inhibition. Similarly, it has been frequently reported that Se could regulate tissue repair by controlling these growth factors. For example, Se added to unripe carica papaya pulp extracts could promote wound repair through early transient expression of *TGFB1* and *VEGFA* [42]. Hydrosoluble nanoselenium (Nano-Se) could promote the action of the FGFR, Wnt, and VEGF signaling pathways to achieve the regeneration of zebrafish tail fins and mouse skin and promote the repair of skin in diabetic mice [43]. In addition, we found that the mRNA expression levels of *TGFB1* and *TGFB3* in the Se group were significantly lower than those in the control group. In this experiment, the cells treated with serum-free basal media for several hours were in an oxidative stress state compared to cells cultured with serum-containing media. Se is a potent antioxidant nutrient due to its ability to act as a variety of key antioxidant components [44]. TGF-β1 is reported to be a pro-oxidant that increases oxidative stress through this growth factor in the condition of Se deficiency [45]. Gastric mucosa has been reported to increase the concentration of TGF-β3 after oxidative stress induced by ischemia/reperfusion [46]. However, changes in TGF-β3 in the Se-deficient state have not been reported so far. Therefore, the underlying mechanisms need further investigation.

The PI3K/AKT signaling pathway is one of the most important pathways regulating cell growth and apoptosis [47]. Studies have found that PI3K/AKT/GSK-3β can act as a key anti-apoptotic signaling pathway, which blocks apoptotic signaling pathways and activates a series of growth signaling pathways leading to promoted cell survival and proliferation [48, 49]. In addition, activation of the PI3K/AKT signaling pathway can regulate downstream BCL-2 and BAX proteins to protect cells from apoptosis [50, 51]. A previous study showed that Se could reduce Pb-induced apoptosis of chicken splenic lymphocytes by activating the PI3K/AKT pathway [52]. In this study, Se activated the LPS-inhibited PI3K/AKT signaling pathway and decreased the BCL-2/BAX protein ratio in a concentration-dependent manner. The present study showed that Se could regulate the PI3K/AKT signaling pathway to reduce cell apoptosis and increase LPS-inhibited BEECs proliferation. Similarly, both Se deficiency and excess impaired the male reproductive system in mice by activating PI3K/AKT-mediated apoptosis and cell proliferation signaling in the testis [53]. And Se could stimulate the proliferation of 3T3-L1 preadipocytes through the activation of the PI3K/AKT signaling pathway [54].

Wnt family members are essential for early embryonic development and pre-implantation endometrial changes [55]. The Wnt/β-catenin signaling pathway plays an important role in cell proliferation and migration during wound healing [56], and some studies have found that Se can regulate cell proliferation through the Wnt/β-catenin pathway [57, 58]. In basal conditions, β-catenin is phosphorylated by a destruction complex of adenomatous polyposis coli (APC), the scaffolding protein Axin, casein kinase 1α (CK1α) and GSK-3β and is then further degraded. Once the Wnt/β-catenin signaling pathway is activated, the destruction complex will be destructed, leading to β-catenin stabilization, cytoplasmic accumulation, nuclear translocation and activating Wnt-dependent gene expression [59]. In a mouse model of Alzheimer’s disease, Se promoted hippocampal neurogenesis via the Wnt signaling pathway [58]. In addition, selenium nanoparticles (SeNPs) could promote the proliferation and differentiation of neural stem cells through the Wnt/β-catenin signaling pathway [60]. In this study, the results of WB and immunofluorescence showed that compared with the LPS group, the expression levels of β-catenin in the co-treatment groups with LPS and Se were significantly increased in a concentration-dependent manner, indicating that Se activated the Wnt/β-catenin signaling pathway. The increased protein levels of c-Myc and Cyclin D1 in the co-treatment groups with LPS and Se were necessary for cell cycle operation [61, 62]. Correspondingly, our cell cycle detection results also demonstrated that Se promoted LPS-inhibited cell cycle operation. Compared with the LPS group, the protein levels of proliferating cell nuclear antigen (PCNA) in the co-treatment groups with LPS and Se were increased in a concentration-dependent manner. PCNA was an essential factor in DNA replication and repair and had been recognized as a marker of proliferation in the clinic [63]. These data indicated that Se could activate the Wnt signaling pathway to promote LPS-inhibited BEECs proliferation.

In the present study, Se supplementation promoted LPS-inhibited proliferation of BEECS, which contributed to promoting the repair of postpartum endometrium and maintaining its normal structure and function. Its potential benefits also include enhancing immune function, promoting antioxidant capacity, and reducing inflammatory response [64–70]. So, applying Se to prevent and treat bovine endometritis holds promise. Due to the differences in many factors, such as geographical environment, the recommended amount of Se supplementation differs among different countries [71–73]. Given that Se contents in cows are typically low and 1 µM is a common level for cows in some areas, we referred to a previous study that ultimately chose 1, 2 and 4 µM to investigate the effect of Se supplementation on uterine repair in cows with endometritis [64, 74, 75]. The results showed that a high concentration (4 µM) of Se was more conducive to promoting cell proliferation than a low concentration (1 µM), we propose that appropriately increasing dietary Se concentrations may be more beneficial to bovine health. On the other hand, given that Se was considered helpful in clinical treatment for renal cell carcinoma [76], developing Se-containing drugs to treat bovine endometritis may also be a new direction. Further research is also needed to fully understand its mechanisms of action. This knowledge will enable the development of effective strategies for applying Se to the cow breeding industry to benefit animal welfare and generate more economic value.

## Materials and methods

### Isolation and culture of BEECs

BEECs were isolated and cultured according to previous articles in our laboratory [77]. In brief, healthy uterine horns of pre-estrus cattle (ovarian stage I, no genital diseases or microbial infections) were collected from a slaughterhouse. A total of 30 cows were used during the studies. These cows were not inseminated and with no signs of genital disease or microbial infection based on cytologic examination and the presence of foul smell, characteristic visual appearance and vaginal discharge [78]. Clinical diseases other than metritis or endometritis, such as milk fever, mastitis, clinical ketosis, and displacement of the abomasum were diagnosed according to farm protocol by experienced farm staff or the herd veterinarian. The estrus cycle was determined according to the method described in a previous study [79, 80]. BEECs were isolated and cultured by enzyme digestion and mechanical scraping. In clean bench, the uterine horns were cut into approximately 3 cm long segments and rinsed clean with phosphate buffer solution (PBS) containing 500 units/mL penicillin and 500 µg/mL streptomycin. The uterine horns were cut longitudinally to fully expose the endometrium. Then, it was rinsed with normal saline and soaked at 4 °C for 30 min. Next, tissues were digested with 1 mg/mL 0.1% protease from Streptomyces griseus (P5147, Sigma, USA), 200 units/mL penicillin and 200 µg/mL streptomycin dissolved in DMEM-F12 (D8900, Sigma, USA) at 4 °C for 12 to 18 h. After that, the endometrium was gently scraped. The harvested endometrium was centrifuged in PBS at 100 × g for 5 min and repeated 3 times. Finally, the isolated cells were placed in DMEM-F12 containing 15% fetal bovine serum (FBS, Gibco, USA), 100 units/mL penicillin and 100 µg/mL streptomycin and inoculated into 25 cm flasks. The cells were cultured at 37 °C and 5% CO_2_. The cell culture medium was changed every 1 to 2 days after cell attachment. Primary endometrial epithelial cells were obtained after approximately 3 to 5 days. Immunohistochemical detection of CK-18 was used to determine the purity of BEECs, and finally, the proportion of BEECs was determined to be greater than 99%. In each subsequent independent experiment, the cells came from a single uterus.

### Experimental design

In this experiment, the BEECs damage model induced by LPS (L6529, Sigma, USA) was established by detecting cell viability, cell apoptosis and the BCL-2/BAX protein ratio. Finally, the suited condition was treated with 10 µg/mL LPS for 24 h. According to the data in our lab [64], the cells in the experimental groups were pretreated with DMEM-F12 containing Se (S5261, Sigma, USA, 1, 2 or 4 µM) for 12 h and then treated with LPS or co-treated with Se. There were six parallel groups: the control group, the LPS group, the Se (1, 2 or 4 µM) plus LPS co-treatment groups, and the Se (4 µM) group.

### Cell viability

Cell viability was detected by using a CCK-8 Cell Counting Kit (A311, Vazyme Biotech, China). In brief, cells were seeded in 96-well plates and grown to 80% confluence in a 5% CO_2_ incubator at 37 °C. Then, the cells were treated with 1, 5, 10, 20 and 30 µg/ml LPS for the indicated time points. CCK-8 was added into each well and incubated for 2 h at 37 °C. The optical density was read at 450 nm using a microplate reader (Tecan, Austria). Six cows were used in this experiment.

### Apoptosis analysis

BEECs were scraped and stained with annexin V-FITC and propidium iodide according to the manufacturer’s instructions of Annexin V-FITC Apoptosis Detection Kit (C1062M, Beyotime, China). In brief, BEECs in 6-well plates after treatment with LPS were harvested and washed with PBS buffer. After being centrifuged at 1000 × g for 5 min, 195 µL of FITC-conjugated annexin V binding buffer, 5 µL of annexin V-FITC and 10 µL of propidium iodide were added. After gentle vortexing, the mixture was incubated for 20 min at room temperature (20 to 25 °C) in the dark. Finally, apoptotic cells were measured by flow cytometry (LSRFortessa, BD Biosciences, USA), and the data were analyzed by CytExpert Software 2.3 (Beckman Coulter, Inc., USA). Cells undergoing early-stage apoptosis are stained with Annexin V-FITC only, and cells at late-stage apoptosis or necrotic cells are stained with both annexin V-FITC and propidium iodide and Annexin V-FITC. Three cows were used in this experiment.

### Cell migration assay

The migration of cells was determined by scratch wound healing assay [13]. Briefly, BEECs grew to 90% confluence in a six-well plate. Cells were wounded with a 200 µL pipette after being pretreated with Se for 12 h. And PBS was used to wash the cells until no cell was visible at the scratch microscopically. Then, images of migrated cells after treatment were captured under an inverted microscope at 100 × magnification at 0 (scratching), 6, 12 and 24 h. Three cows were used in this experiment.

### Cell cycling analysis

Detection of the cell cycle and apoptosis by flow cytometry was carried out using a cell cycle and apoptosis analysis kit (C1052, Beyotime, China). The BEECs were harvested by gentle trypsinization after treatment. Then, the cells were washed twice with cold PBS, and collected by centrifugation, and fixed in 70% ethanol (4 °C) for 24 h. Subsequently, the fixed cells were washed twice with cold PBS and resuspended with RNase A and propidium iodide (C1052, Beyotime, China) in the dark at 37 °C for 30 min. Flow cytometry was performed immediately, and the data were analyzed by FlowJo software 7.6 (BD Biosciences, USA). Three cows were used in this experiment.

### EdU proliferation assay

Proliferation was performed using the BeyoClick™ EdU Cell Proliferation Kit with Alexa Fluor 488 (C0071S, Beyotime, China) according to the manufacturer’s protocol. In brief, BEECs after treatment were incubated with 10 µM EdU for 3 h at 37 °C, followed by fixing with 4% paraformaldehyde for 15 min and treating with 0.4% Triton X-100 (ST797, Beyotime, China) for 15 min at 37 °C. Next, the cells were washed three times using 3% BSA in PBS, cultured with 0.5 mL of Click Reaction Mixture for 30 min in the dark and the nuclei were stained with Hoechst 33,342 for 15 min at room temperature. The fluorescence images were observed by the fluorescence microscope (Leica TCS SP8, Leica Corporation, Germany). Three fields of view were randomly selected in each sample, and the percentage of EdU-positive cells was determined. The number of cells in each field of view was counted independently. Three cows were used in this experiment.

### RNA extraction and quantitative real-time polymerase chain reaction (qPCR)

Total RNA was isolated from BEECs using TRIzol reagent (ET111, TRAN, China). A Nanodrop 2000 spectrophotometer (Thermo, USA) was used to detect the quantity and purity of the extracted RNA. Each sample was tested three times, and the OD_260nm_/OD_280nm_ ratio was between 1.8 and 2.1. The cDNA was synthesized from 900 ng extracted RNA from each sample using a HiScript II 1st Strand cDNA Synthesis Kit (R211-1, Vazyme, China) according to the manufacturer’s instructions. Primer sequences are listed in Table 1 according to a previous paper [13] and were purified and sequenced by TsingKe Biotech, China. The sequence results were analyzed using BLAST and compared to the GenBank database. The real-time PCR reaction system included 10 µL ChamQ SYBR qPCR Master Mix, 0.4 µL of each primer (10 µM), and 2 µL of cDNA template in a final volume of 20 µL per reaction (Q311, Vazyme, China). The qPCR was performed at 95 °C for 30 s, followed by 40 cycles of amplification at 95 °C for 10 s and 60 °C for 30 s by using the 7500 Real-time PCR system (Applied Biosystems, Life Technologies, Corp., USA). The results were repeated at least three times independently from three different pools of templates and were calculated using the 2^−△△Ct^ method. The mRNA level of *ACTB* was used as an internal control. Six cows were used in this experiment.

**Table 1:** Primer sequences for real-time PCR amplification

Gene	Primer	Sequence (5’ $\to$ 3’)	Product (bp)	Accession number
*TGFB1*	Forward	CGAGCCCTGGACACCAACTA	137	NM_001166068.1
	Reverse	AGGCAGAAATTGGCGTGGTA		
*TGFB3*	Forward	CTGTGCGTGAATGGCTCTTG	153	NM_001101183.1
	Reverse	CATCATCGCTGTCCACACCT		
*VEGFA*	Forward	GACCCTGGTGGACATCTTCC	127	NM_001316992.1
	Reverse	CACACAGGGCACACACTCC		
*CCN2*	Forward	AGCTGACCTGGAGGAGAACA	139	NM_174030.2
	Reverse	GTCTGTGCACACTCCGCAGA		
*ACTB*	Forward	CATCACCATCGGCAATGAGC	156	NM_173979.3
	Reverse	AGCACCGTGTTGGCGTAGAG		

### Protein extraction and western blotting

The nuclear and total proteins were extracted using a Nuclear Protein Extraction kit (P0027, Beyotime, China) and RIPA Lysate Buffer (P0013B, Beyotime, China) according to the manufacturer’s instructions. Protein concentration was determined using the BCA Protein Assay Kit (Beyotime Biotech, Nantong, China). Equal protein concentrations were separated by 12% sodium dodecyl sulfate‒polyacrylamide gel electrophoresis and transferred onto polyvinylidene difluoride (PVDF) membranes (Millipore, Bedford, MA, USA). Then, the membranes were blocked for 2 h with TBST (50 mmol/L Tris, pH 7.6, 150 mmol/L NaCl, and 0.1% Tween 20) containing 5% nonfat milk. After washing three times in TBST, the membranes were cut prior to hybridization with primary antibodies diluted in TBST overnight at 4 °C. The antibodies against PI3K (# 4292), p-PI3K (# 4228), AKT (# 4691), p-AKT (# 4060), Cyclin D1 (# 2978), c-Myc (# 5605) and β-actin (# 4970) were purchased from Cell Signaling Technology (Danvers, MA, USA); PCNA (# 10205-2-AP) and BAX (# 50599-2-Ig) were purchased from Proteintech Group (Wuhan, China); BCL-2 (# sc-7382) was purchased from Santa (Nanjing, China); and GSK-3β (# ab75814), p-GSK-3β (# ab32391) and β-catenin (# ab32572) were purchased from Abcam (Shanghai, China). Then, the membranes were washed and incubated with horseradish peroxidase-conjugated secondary antibodies for 1 h at room temperature and then washed three times for 10 min. The immunoreactive bands were incubated in enhanced chemiluminescence (Millipore, Shanghai, China) and exposed to the ChemiScope5300Pro CCD camera (Clinx Science Instruments, Shanghai, China). The data were analyzed using ImageJ software. Each group was set up with three replicates. Three cows were used in this experiment.

### Immunofluorescence staining

BEECs grew on coverslips in 24-well cell culture plates. After treatment, the cells were fixed with 4% paraformaldehyde for 15 min at room temperature. Then, 0.4% Triton X-100 (ST797, Beyotime, China) was used to penetrate the cellular membranes for 15 min at 37 °C after washing with PBS. The cells were washed in PBS and blocked with 5% bovine serum albumin (BSA) for 2 h at 37 °C. Then, the cells were incubated with anti-β-catenin (diluted in 5% BSA) at 4 °C overnight. The next day, the cells were incubated with a FITC-conjugated secondary antibody (A0423, Beyotime, China) for 1 h at 37 °C. The nuclei were stained with DAPI Staining Solution (C1005, Beyotime, China) for 15 min at 37 °C in the dark. Finally, the BEECs were observed by using a fluorescence microscope. Three cows were used in this experiment.

## Statistical analysis

All data were presented as the mean ± standard error of the mean (SEM) of at least three independent experiments. Statistical analysis of the results was performed using the independent t-test and one-way ANOVA using IBM SPSS Statistics 25.0 (IBM, USA). A value of P less than 0.05 was considered to be significant statistically.

## Conclusions

The present study demonstrated that Se could protect BEECs against LPS-induced damage and promote cell proliferation and migration in vitro. This effect was possibly regulated by increasing the gene expressions of growth factors (*TGFB1*, *TGFB3* and *VEGFA*) and activating the PI3K/AKT and Wnt/β-catenin signaling pathways.

### Electronic supplementary material

Below is the link to the electronic supplementary material.

Supplementary Material 1

## Data Availability

The datasets analyzed during the current study are available from the corresponding author on reasonable request.

## Abbreviations

BEECs: Bovine endometrial epithelial cells
Se: Selenium
LPS: Lipopolysaccharide
PI3K: Phosphatidylinositol 3-kinase
AKT: Protein kinase B
*E. coli*: *Escherichia coli*
PBS: Phosphate buffer solution
PVDF: Polyvinylidene difluoride
VEGF: Vascular endothelial growth factor
CTGF: Connective tissue growth factor
TGF-β: Transforming growth factor-beta
ECM: Extracellular matrix
GSK-3: Glycogen synthase kinase-3
APC: Adenomatous polyposis coli
CK1α: Casein kinase 1α
SeNPs: Selenium nanoparticles
PCNA: Proliferating cell nuclear antigen
