# Supplementary material for: The effect of selenium on the proliferation of bovine endometrial epithelial cells in a lipopolysaccharide-induced damage model
Source: BMC Vet Res. 2024 Mar 18;20:109. doi: 10.1186/s12917-024-03958-4 (PMC10946195; doi:10.1186/s12917-024-03958-4)

Pages 1 to 3, 4 to 12, and 13 to 18 are the original blots of Figure 1C, Figure 6A, and Figure 6B in manuscript (three replicate blots), respectively.

1-BCL-2

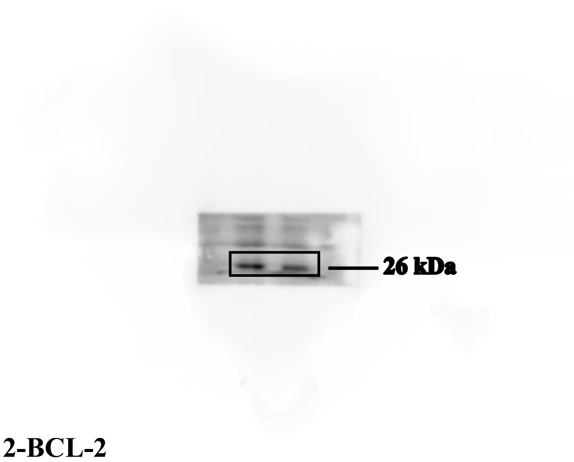

2-BCL-2

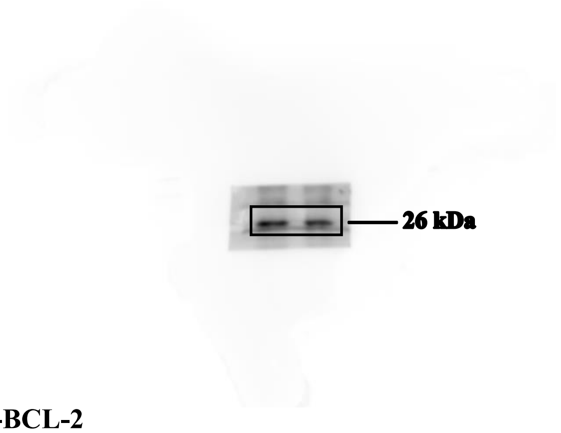

3-BCL-2

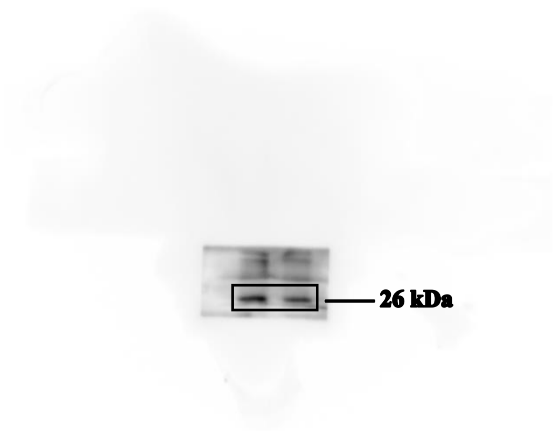

1-BAX

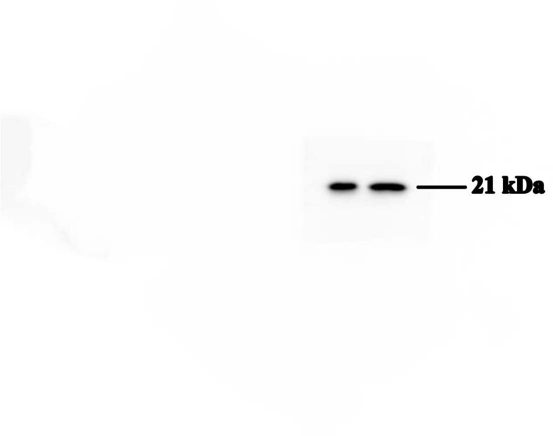

2-BAX

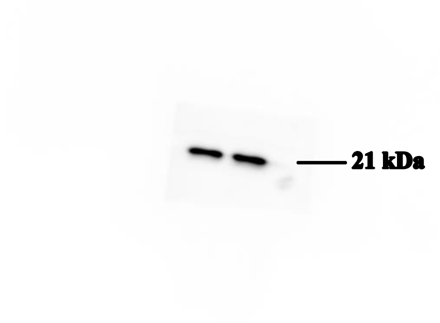

3-BAX

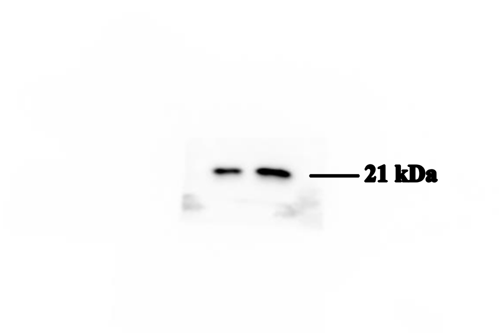

1-β-actin

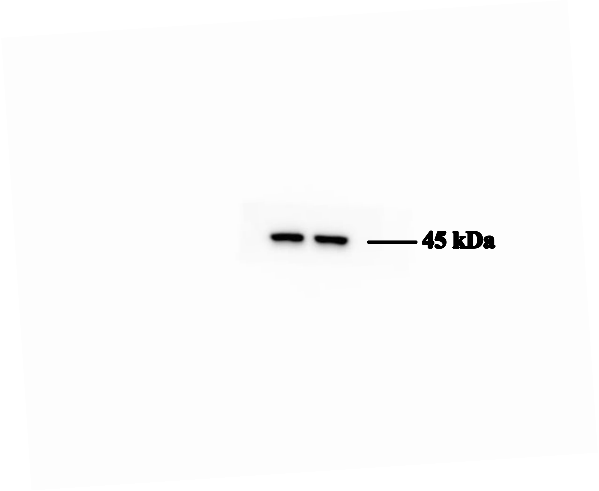

2-β-actin

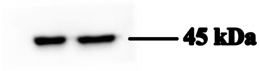

3-β-actin

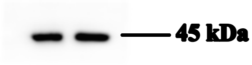

1-PI3K

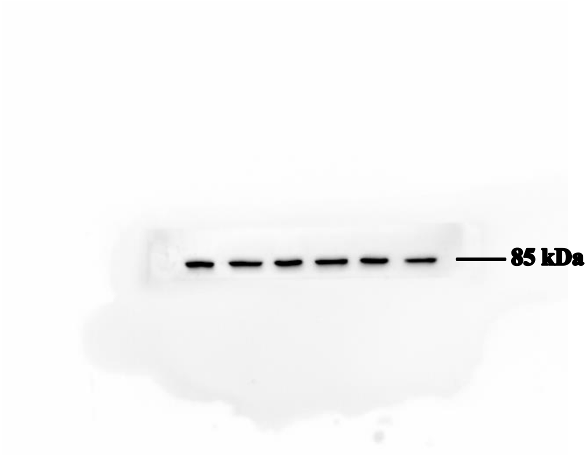

2-PI3K

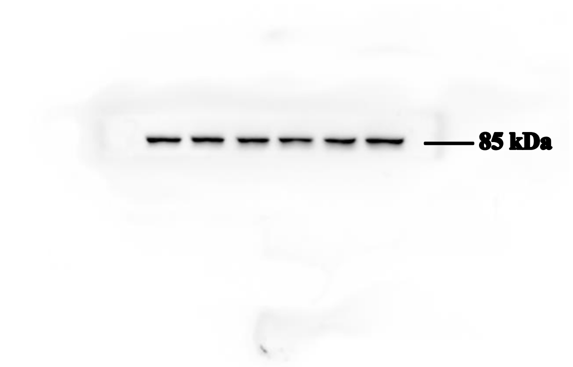

3-PI3K

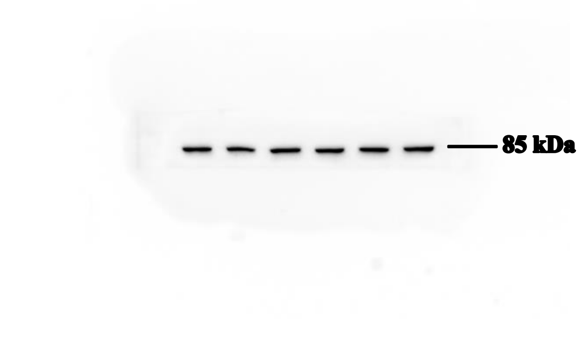

1-p-PI3K

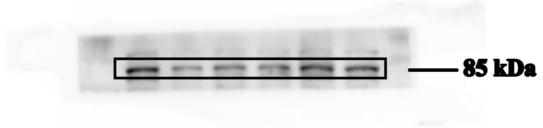

2-p-PI3K

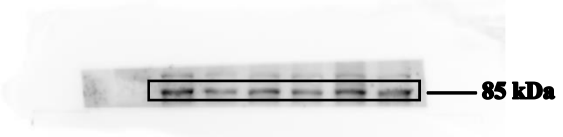

3-p-PI3K

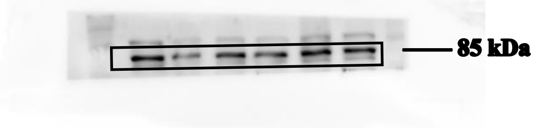

1-AKT

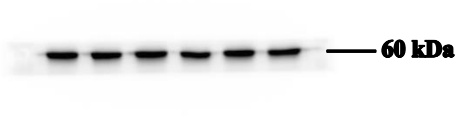

2-AKT

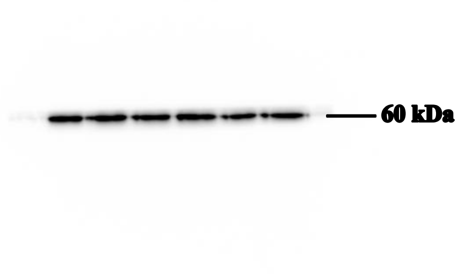

3-AKT

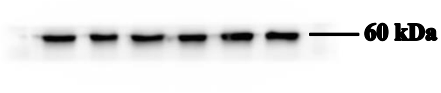

1-p-AKT

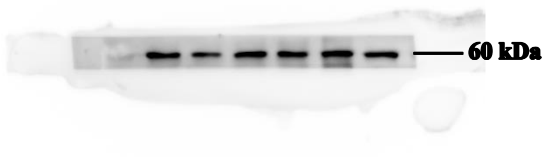

2-p-AKT

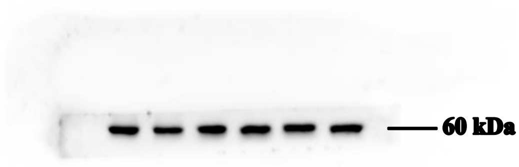

3-p-AKT

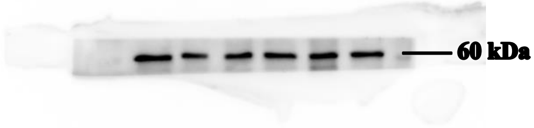

1-GSK-3β

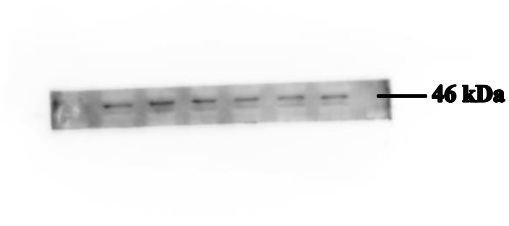

2-GSK-3β

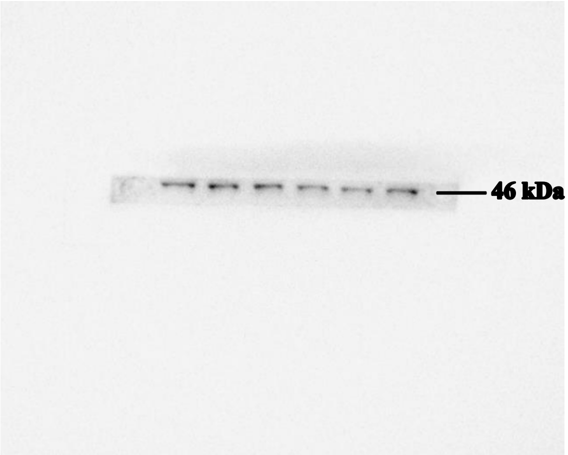

3-GSK-3β

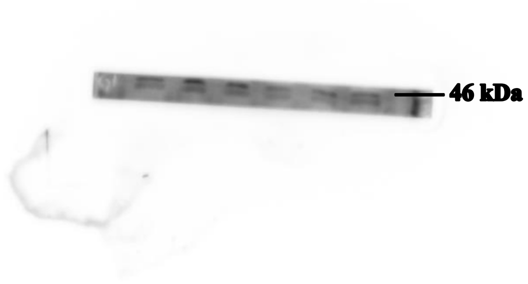

1-p-GSK-3β

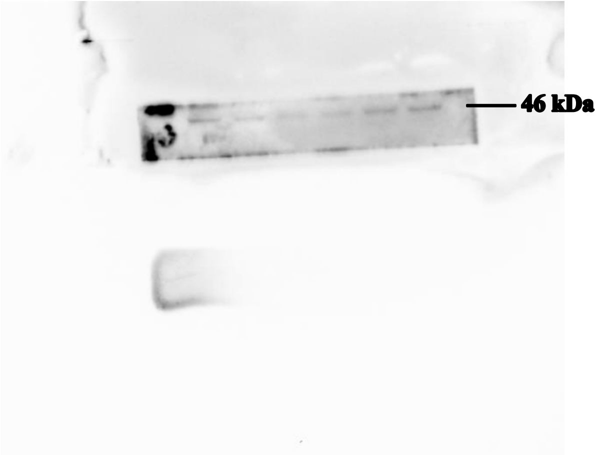

2-p-GSK-3β

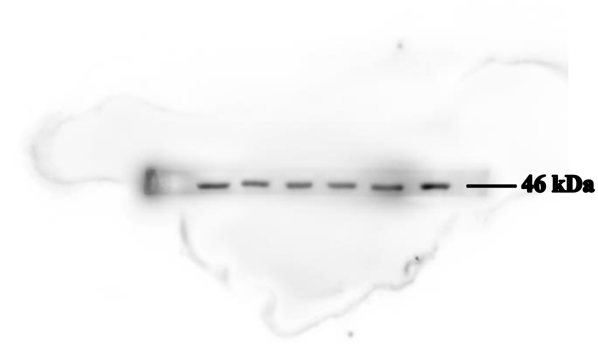

3-p-GSK-3β

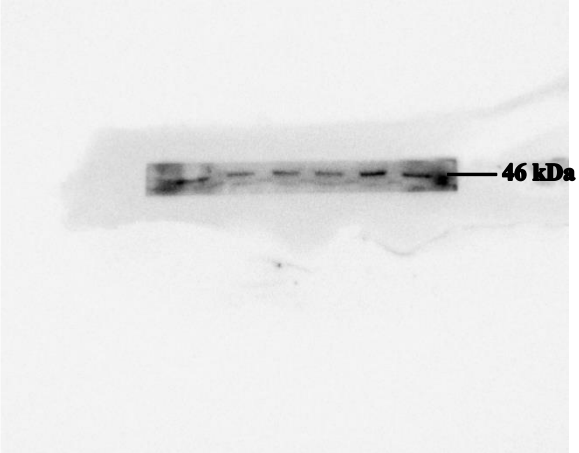

1-BAX

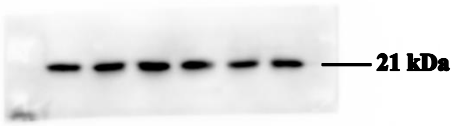

2-BAX

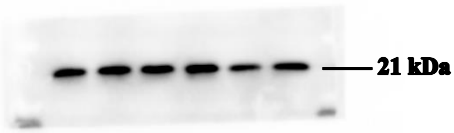

3-BAX

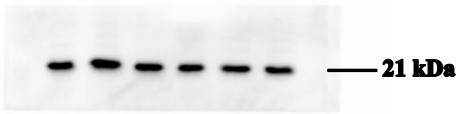

1-BCL-2

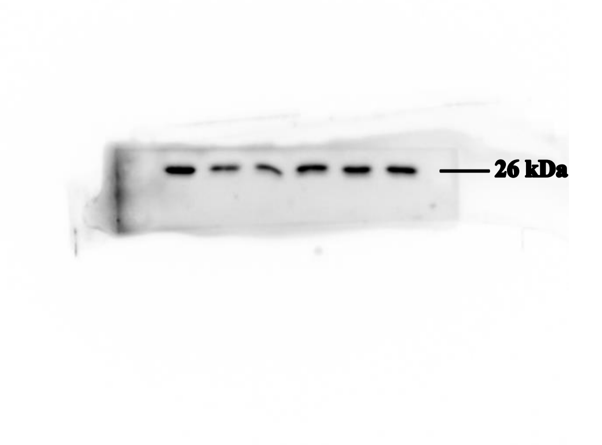

2-BCL-2

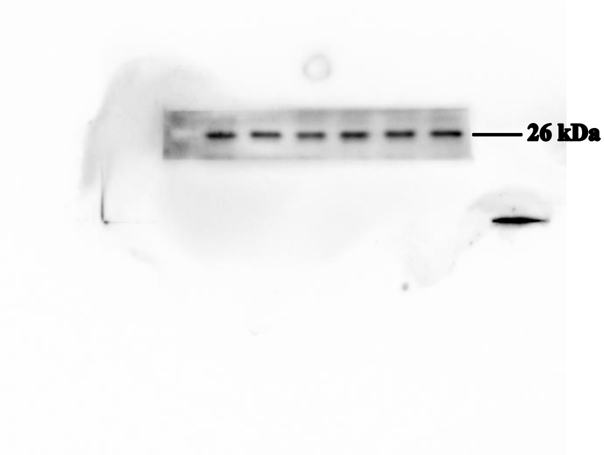

3-BCL-2

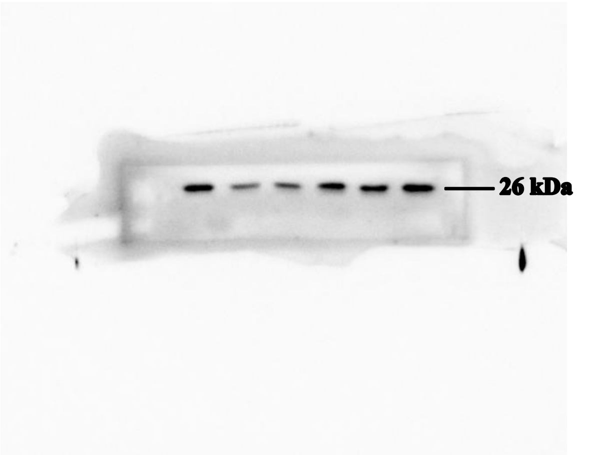

1-β-actin

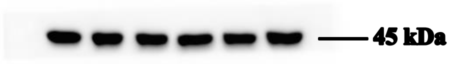

2-β-actin

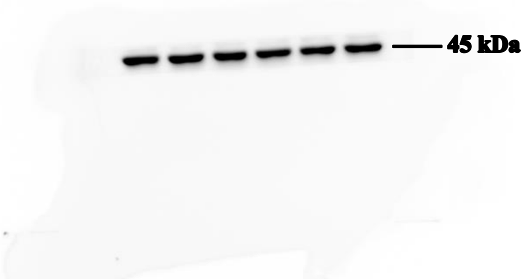

3-β-actin

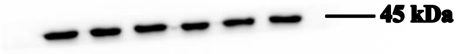

1-β-catenin (nucleus)

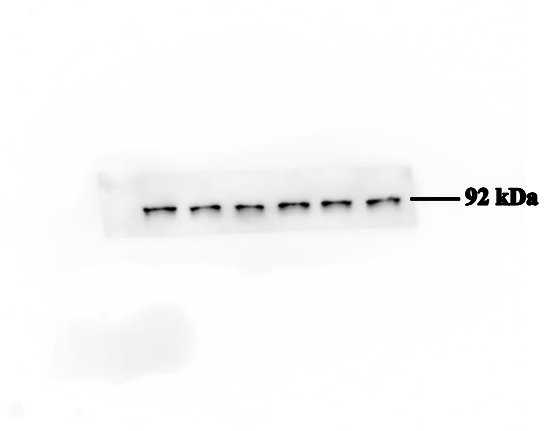

2-β-catenin (nucleus)

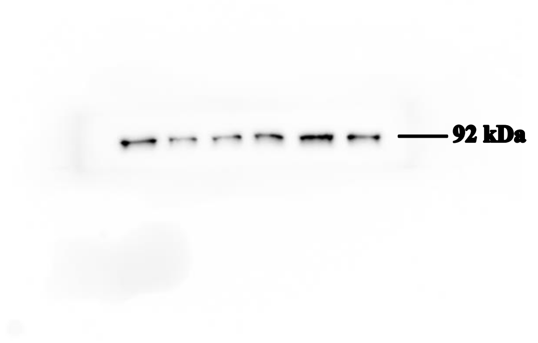

3-β-catenin (nucleus)

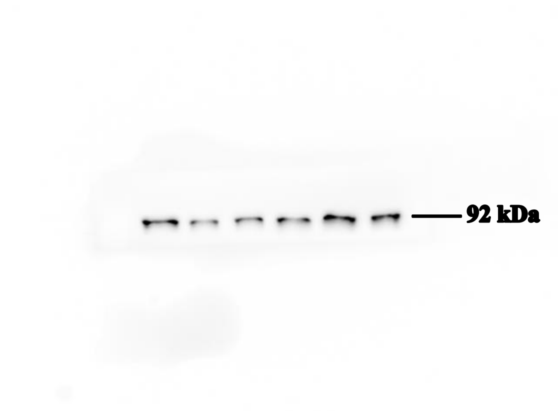

1-Lamin B1

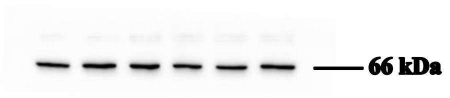

2-Lamin B1

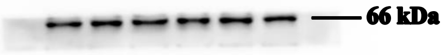

3-Lamin B1

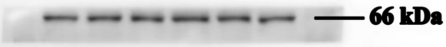

1-c-Myc

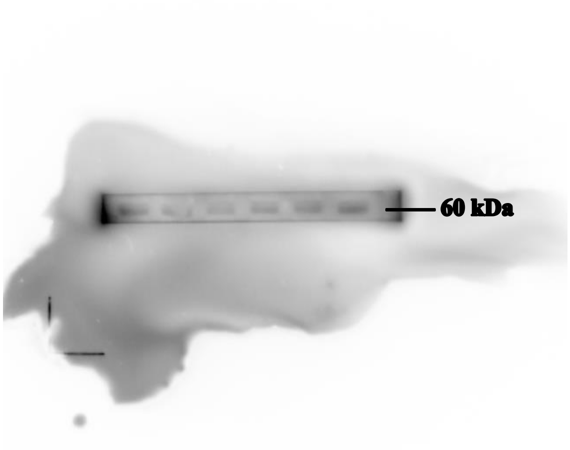

2-c-Myc

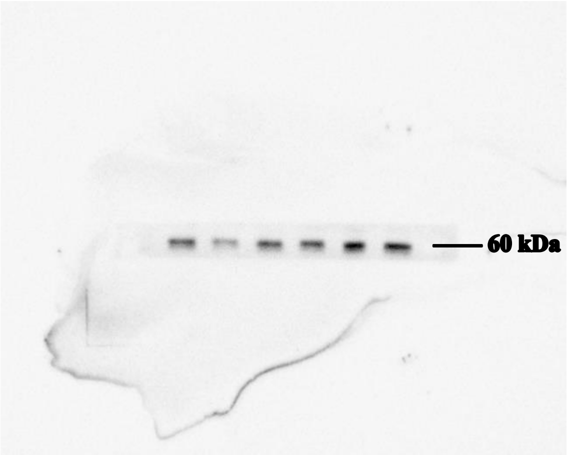

3-c-Myc

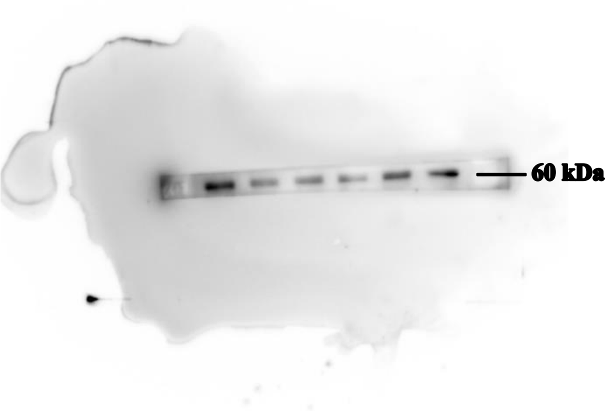

1-Cyclin D1

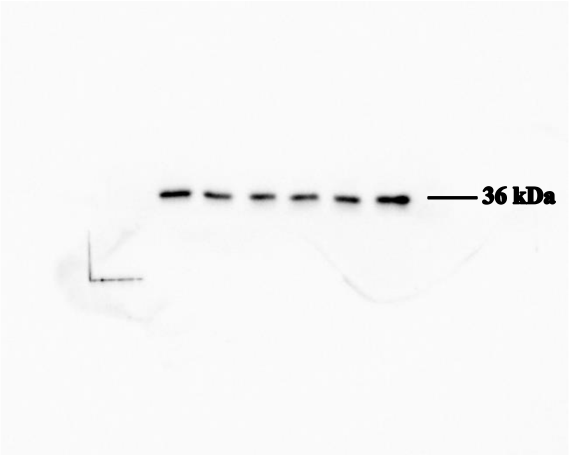

2-Cyclin D1

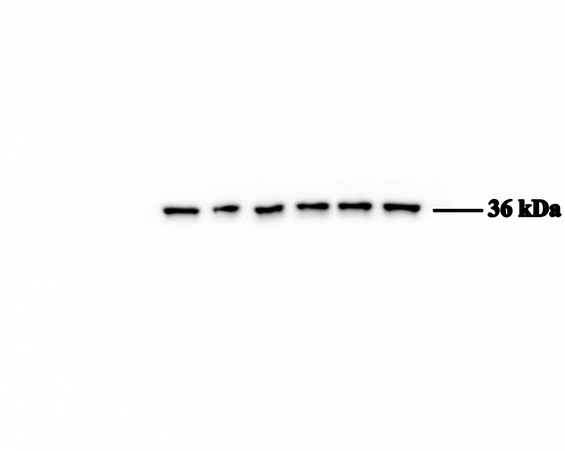

3-Cyclin D1

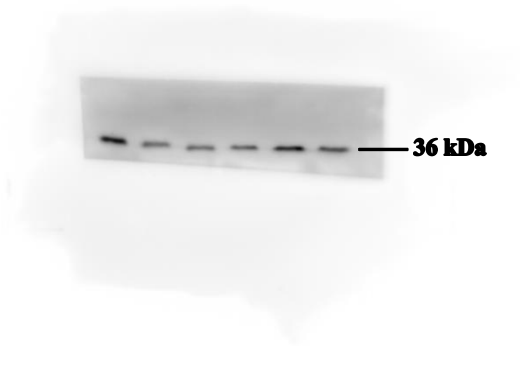

1-PCNA

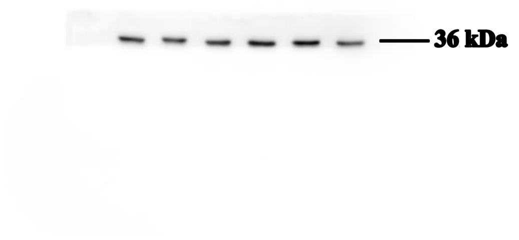

2-PCNA

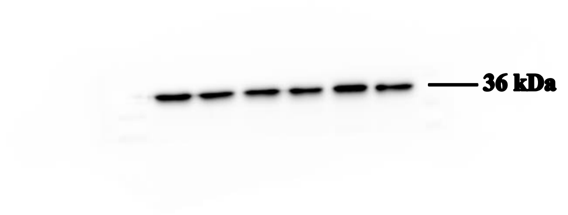

3-PCNA

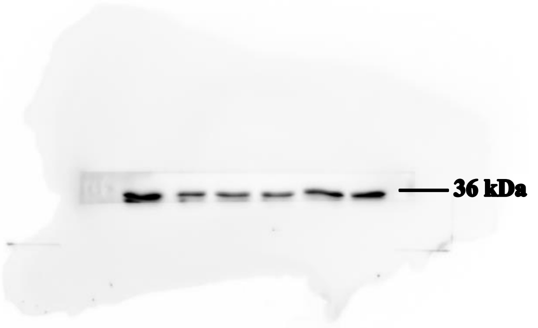

1-β-actin

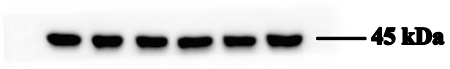

2-β-actin

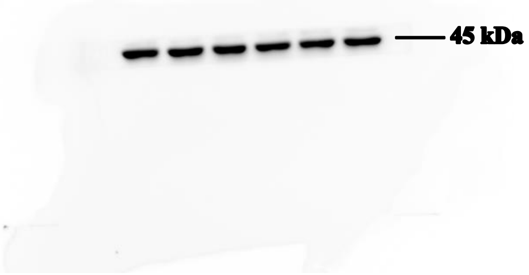

3-β-actin

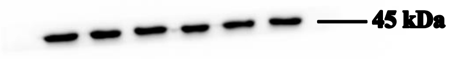

Supplement: Supplementary file 1 — Supplementary Material 1 [file 12917_2024_3958_MOESM1_ESM.pdf]
